# Atomap: a new software tool for the automated analysis of atomic resolution images using two-dimensional Gaussian fitting

**DOI:** 10.1186/s40679-017-0042-5

**Published:** 2017-02-13

**Authors:** Magnus Nord, Per Erik Vullum, Ian MacLaren, Thomas Tybell, Randi Holmestad

**Affiliations:** 1 Department of Physics, Norwegian University of Science and Technology, Trondheim, Norway; 2 SUPA, School of Physics and Astronomy, University of Glasgow, Glasgow, UK; 3 Materials and Chemistry, SINTEF, Trondheim, Norway; 4 Department of Electronics and Telecommunications, Norwegian University of Science and Technology, Trondheim, Norway

**Keywords:** Quantitative STEM, Strain mapping, Image processing, Oxygen octahedral distortion, Non-rigid registration

## Abstract

Scanning transmission electron microscopy (STEM) data with atomic resolution can contain a large amount of information about the structure of a crystalline material. Often, this information is hard to extract, due to the large number of atomic columns and large differences in intensity from sublattices consisting of different elements. In this work, we present a free and open source software tool for analysing both the position and shapes of atomic columns in STEM-images, using 2-D elliptical Gaussian distributions. The software is tested on variants of the perovskite oxide structure. By first fitting the most intense atomic columns and then subtracting them, information on all the projected sublattices can be obtained. From this, we can extract changes in the lattice parameters and shape of A-cation columns from annular dark field images of perovskite oxide heterostructures. Using annular bright field images, shifts in oxygen column positions are also quantified in the same heterostructure. The precision of determining the position of atomic columns is compared between STEM data acquired using standard acquisition, and STEM-images obtained as an image stack averaged after using non-rigid registration.

## Background

Scanning transmission electron microscopy (STEM) together with correction of geometric aberrations in the probe forming optics allows routine acquisition of atomic resolution images with sub-Å resolutions [1, 2]. These images contain a wealth of information about the crystal structure of a material. Specifically:

1. The position of the atom columns in high angle annular dark field (HAADF) images can be determined quantitatively and used for structure solution [3–6], and the determination of the structure of defects [7].
2. The position of the atom columns can be used to get local changes of lattice parameters [8].
3. In HAADF-STEM, the intensity of an atomic column is related to the atomic number of the elements in the atomic columns and the number of atoms in the columns [9]. Simulations are often needed to interpret the intensity quantitatively as there are complicating effects from sample orientation [10], material phase [10], defects [11], and strain [11] in the material. By combining HAADF-STEM with simulations, one can extract compositional and thickness information, in some cases even counting all the atoms [10].
4. Even information about the structure parallel to the electron beam can be inferred from the shape of the columns [13].

With the wealth of information in these images, having robust and quantitative methods for analysing them is just as important as acquiring them.

The work in this paper is performed using the perovskite structure, although the principles could be used for many other crystal structures. The perovskite structure derives from the mineral CaTiO$_3$ and the generic formula of a perovskite oxide is ABO$_3$; the simplest form of the structure is a primitive cubic structure with the A-sites at the corners, the B-sites at the body center, and the O-sites at the face centers of each cell.

IIn addition to the aforementioned STEM-HAADF imaging, which is best for heavier elements, it is also important to be able to image and quantify the positions of lighter elements. For example, in perovskite oxides it is vital to be able to accurately map the position of the oxygen columns [14]. In recent years, STEM imaging using either bright field (BF) [8, 15, 16] or annular bright field (ABF) [17, 18] conditions has been useful for revealing oxygen atom columns in such oxides.

Of special interest is the oxygen structure across interfaces in heterostructures [19], since this is very hard to probe with other techniques. In perovskite oxides, the oxygen positions can be used to infer the oxygen octahedral tilting pattern [20], which is important for understanding the macroscopic functional properties of the material, and may well be affected by constraints from coherent interfaces [8, 18].

A commonly used method for quantifying changes in lattice parameter is geometrical phase analysis (GPA) [21]. GPA is based on Fourier transforming atomic resolution images and placing masks around two non-collinear Bragg spots. Historically, this method has been used with high-resolution TEM (HRTEM) [21, 22]. However, with the advent of STEM aberration correctors, it has also seen extensive use on STEM-images [5, 6, 23]. While this is a fast and easy way to calculate deformation of a lattice, it can introduce artefacts [24] and the spatial resolution is limited to 1 unit cell [23] due to it being based on Fourier transforming the image data. Ideally, it would be preferable to use real space methods, which do not require the use of Fourier transforms. One possibility is to use the center of mass for each bright column which is usually robust [25], but this has the limitation that it only gives the center positions of the atomic columns. Alternatively, the fitting of a 2-D Gaussian to the bright column will give the width, ellipticity, amplitude, and more precise center position [25]. However, for this to work successfully, reasonable initial values are needed.

Several software tools for real space analysis exist: Ranger [26], qHAADF [27], iMtools [28], StatSTEM [29], and Oxygen octahedra picker [30]. These methods have been used in several works: using center of mass combined with principal component analysis (PCA) [13], pattern matching [31], iMtools using 2-D Gaussians [4, 32], and MATLAB with the Image Processing Toolbox [10]. Recent work has also used computer vision-based techniques to characterize the local structure [33].

Since STEM-images can show several thousand atomic columns, automation is an important aspect for analysis methods. Ideally, such methods should require as little manual input as possible, since this allows analysis of large images containing several thousand atomic columns. This is important for three reasons: (i) the more information, the better, (ii) researcher time is valuable, computing time is cheap, (iii) large sample sizes allows for a more statistical approach to data analysis. The automation should ideally do the peak finding and position refinements. In addition, it should also construct relations between the atoms. For example, for an image of a monocrystalline material, the atoms belonging to the same monolayer should automatically be identified. This enables rapid analysis of parameters like distances between monolayers, and changes in lattice parameters.

In addition, this framework should be free and open source [34]. This avoids the processing steps being hidden in a “black box”, and allows for other researchers to improve and extend the functionality.

In this work, we present Atomap, a new free and open source software package for automatic analysis of the position and shape of atomic columns in STEM-images. Using a variety of peak finding and position refinements, even light elements, such as oxygen, can be accurately quantified. We start by outlining the method by showing the different processing steps on a SrTiO$_3$ (STO) substrate. Next, the method is applied to extract structural information from different perovskite oxide heterostructures. In particular, the position of sublattices in the crystal structure, the shape of atomic columns, and superstructures in oxygen atomic planes are determined.

## Computational and experimental methods

The focus of this work is the analysis of atomic resolution STEM-images of perovskite oxides. As mentioned earlier, these materials are in the form of ABO$_3$. The A-site is a larger cation like strontium or lanthanum, the smaller B-site is typically a transition metal like manganese or titanium, and the O is oxygen. A-site cations are usually the heaviest element in the structure, the B-site cations the second heaviest, and oxygen the lightest. The heterostructures studied were La$_{0.7}$Sr$_{0.3}$MnO$_3$ (LSMO) on LaFeO$_3$ (LFO) on (111)-oriented Nb-doped STO and LSMO on (111)-oriented Nb-doped STO. TEM samples were prepared as thin sections perpendicular to the [1$\overline{1}$0]-direction of the STO. Deposition [35, 36] of the films and the preparation of the TEM specimens [37] are described in more detail elsewhere.

**Fig. 1:**
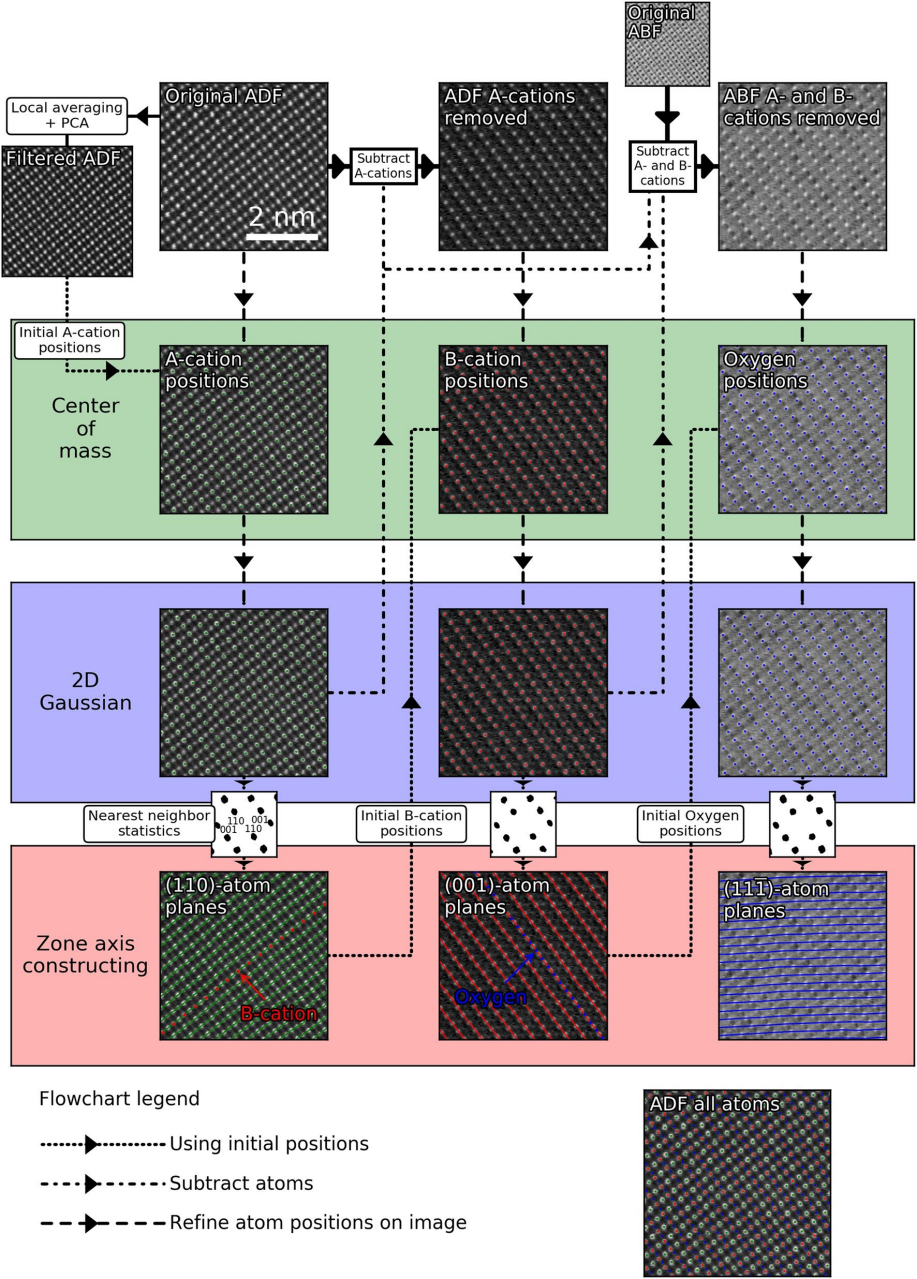
Processing steps for locating and fitting 2-D Gaussians to every atomic column in a perovskite oxide using STEM-ADF and STEM-ABF data acquired with the electron beam parallel to the [1$\overline{1}$0] direction

An example of a typical STEM image is shown in Fig. 1 (top left). The first aim of the method is to extract the position and shape for all the different atomic columns in these kinds of images. Second, we want to find the relations between the different atomic columns. In essence, the process of fitting one sublattice can be summed up in three steps: (i) Find the positions of all the atomic columns you want to examine. (ii) Refine the positions using center of mass until they are close enough for the 2-D Gaussian fitting to work robustly. (iii) Fit the atomic columns using a 2-D elliptical Gaussian function $I(x,y)$. This is defined by the following:

1 $$\begin{aligned} I(x,y)&= I_0 + A\exp {\left( -\left( a(x-x_0)^2-2b(x-x_0) (y-y_0)+c(y-y_0)^2\right) \right) } \nonumber \\ a&= \frac{\cos ^2{\theta }}{2\sigma ^2_x}+\frac{\sin ^2 {\theta }}{2\sigma ^2_y}\nonumber \\ b&= -\frac{\sin {2\theta }}{4\sigma ^2_x}+\frac{\sin {2\theta }}{4\sigma ^2_y}\nonumber \\ c&= \frac{\sin ^2{\theta }}{2\sigma ^2_x}+\frac{\cos ^2 {\theta }}{2\sigma ^2_y} \end{aligned}$$

where ${I}_0$ is the background, *A* the amplitude, ${x}_0, {y}_0$ the center positions, $\sigma _x, \sigma _y$ the standard deviations, and $\theta$ the rotation. The background $I_0$ is set to the minimum intensity value of the region around the atomic column. This way of setting the background value is easy and robust. However, it has some drawbacks in that a single pixel with low value due to some kind of artefact can lead to the background varying greatly between the different atomic columns. One way of improving this is by having the background as a parameter while fitting the 2-D Gaussians; however, this reduces the robustness as the chance of poor fitting increases. Therefore, in this work, the simpler minimum value method was used, as it worked well in practice. More advanced forms of background subtraction will be implemented in Atomap in the future.

Additional sublattices are found by having *a priori* crystallographic knowledge on where they are located in relation to the first sublattice, as explained below.

### Initial positions and refinements

To exemplify this, we show the procedure to find the positions of all sublattices in an STO crystal projected along the [1$\overline{1}$0]-direction. While this demonstrates the use of this method on a specific crystal structure along a specific projection, the software should work for any kind of atomic structure or projection, as long as the atomic columns are clearly resolved. Comments on how to adapt this for other structures and projections are outlined in "Adapting for other structures and projections".

#### A-cations

First, the original ADF image (Fig. 1, top left) is filtered. This involves doing a local averaging, where a Gaussian convolution of the image is made and subtracted from the original image. Next, 1-D PCA [38] denoises the modified image to reduce random noise. The outcome is a filtered ADF image with smaller intensity variations and less noise. The A-cations in the filtered ADF image are located using a peak finding method which finds the most intense local features, where each feature has to be separated by a minimum distance. This filtered ADF image is only used for the initial peak finding, all subsequent position refinement is done using the original, unfiltered, ADF image.

Next, these initial A-cation positions are refined using the original ADF image. This is done by finding the center of mass for a circular area centered at the current position with a radius of 40% of the distance to the closest A-cation neighbor. The result of this refinement is shown in the “A-cation positions” image in Fig. 1. These A-cation positions are used as the initial values for fitting 2-D Gaussians to every A-cation atomic column in the original ADF image.

The refined positions of the A-cations are the input parameters to further study the average 2-D atomic arrangement of the structure. For each A-cation, the distance and direction to the ten nearest neighbors are calculated. Next, using a similar peak finding process as explained earlier, all the repeating nearest neighbors are found. The “Nearest neighbor statistics” to the bottom left in Fig. 1 shows the real space nearest neighbor distance and direction, which gives information similar to an FFT: the average 2-D arrangement of atoms in a small repeating unit of this specific projection of the 3-D crystal structure. Using different planes visible in the image (i.e., perpendicular to the beam direction), atom columns which belong to the same atom planes are grouped. These atom planes are defined in the software by the vector perpendicular to the trace of the plane in the image. Thus, the atom planes shown in Fig. 1 (bottom left) are the (110) atom planes. The traces of these atom planes run in the [001]-direction.

#### B-cations

Fitting Gaussians to the B-cations is more challenging, due to the A-cations being more intense. To get robust fitting of the B-cations, the intensity from the A-cations is removed from the original ADF image before starting the B-cation fitting. This is done by subtracting the 2-D Gaussians fitted to the A-cations. The original ADF image with the A-cations subtracted is shown in the top center of Fig. 1. This leaves the B-cations as the most intense feature in the ADF image.

The initial positions of the B-cation atomic columns are placed between each A-cation pair in the (110) atom planes. This is shown for one A-cation atom plane in Fig. 1 (bottom left), with the B-cation initial positions marked with red circles.

With the initial B-cation positions and the ADF image with the A-cations removed, the B-cation positions are refined using center of mass the same way as for the A-cations. The refined positions are used as the initial values when doing Gaussian fitting for the B-cations. The 2-D repeating units and atomic planes for the B-cations are constructed in the same way as for the A-cations. This process is shown in the middle column of Fig. 1, where the resulting B-cation (001) atom planes are shown.

#### Oxygen

The rightmost column in Fig. 1 shows how the oxygen positions are determined. The oxygen initial positions are placed between each pair of B-cations in the (001) atom planes, shown with the blue circles in the lower center image in Fig. 1. In ADF imaging, the oxygen is much less intense compared to the heavier A and B cations, so ABF imaging is utilized. Such an image is shown to the top right in Fig. 1 (original ABF), which has been acquired simultaneously with the ADF image. In the ABF image, the oxygen is visible, but still the least intense of the atomic columns. Using the initial A and B cation positions from the ADF image, 2-D Gaussians are fitted to the A and B cations in the ABF image and subtracted. The image contrast is further inverted, to create a modified ABF image where the oxygen columns are the most intense features in the image. Using this modified ABF image and the initial oxygen positions, the positions are refined using the center of mass, further refined using 2-D Gaussians, 2-D repeating units found, and the atomic planes constructed.

The end result gives the location of all the atom columns in the image, as shown to the lower right in Fig. 1.

### Finding distances between atomic columns

**Fig. 2:**
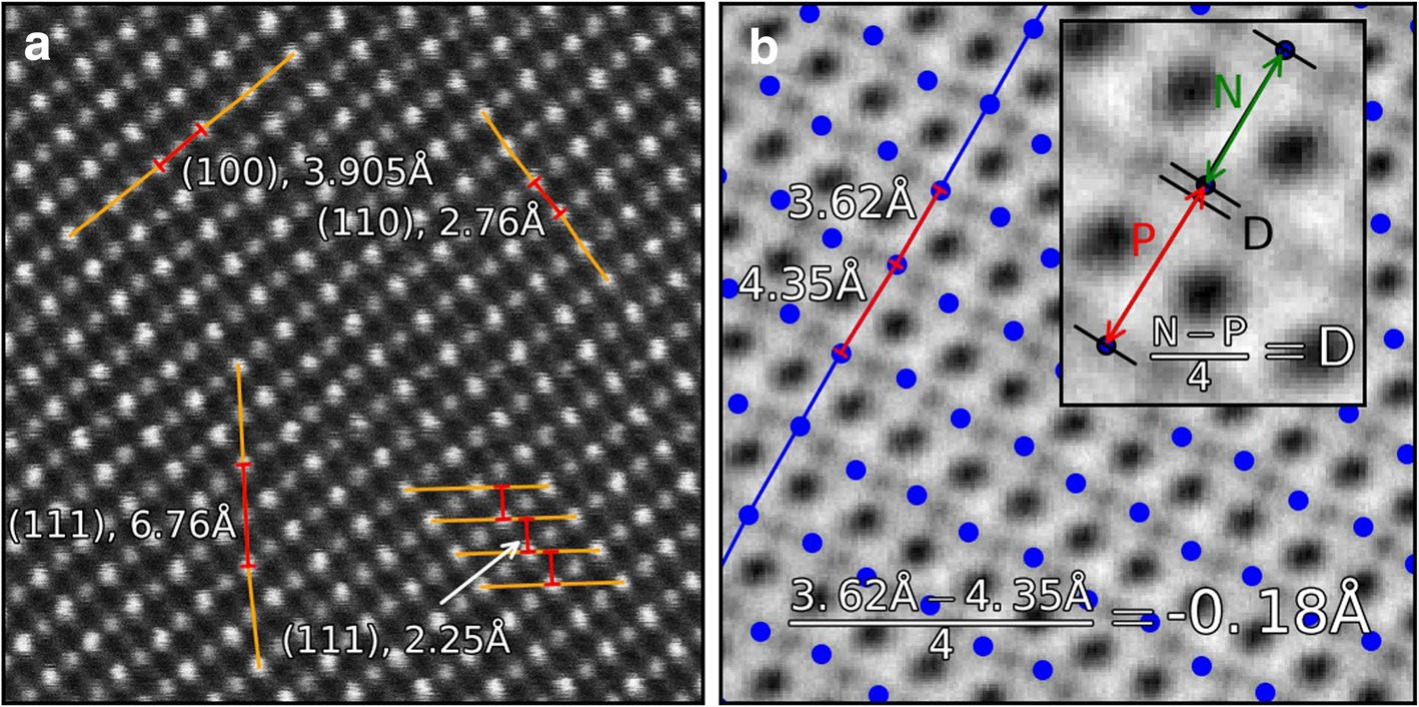
Quantifying distances between atomic planes. **a** Distances between Sr atomic planes in different directions. Showing how finding the distance between atomic planes is straightforward for the [001] and [110] directions, but not for the [111] direction. **b** Calculating displacement (*D*) from a centrosymmetric position between atomic columns, here shown on oxygen columns. The *inset* shows how the distance difference is calculated for oxygen atoms in the [001]-direction for an LFO film. The distance to the next (*N*) and previous (*P*) atom is calculated, as shown with the *green* and *red* *double headed arrows*

Having an accurate position for all the atomic columns is the first step toward making measurements of distances between columns or interplanar spacings. Having already grouped the atom columns into atomic planes, it is trivial to find the spacings in the (001)- and (110)-planes. The distances between neighboring atomic columns in the (001)-planes correspond to the (110) interplanar spacing as these are orthogonal (Fig. 2a). Similarly, the interplanar distances for the (110)-planes are found using the distances between the atomic columns in the (001)-planes. The case is less straightforward for (111)-planes, as neighboring atomic columns (of the same cation type) along the orthogonal (11$\overline{2}$)-plane will be three monolayers apart. The interplanar spacing is the distance between one monolayer and its neighbor. To find this, a line is interpolated through the atomic columns in a (111)-plane. From an atomic column in the neighboring (111)-plane, the shortest distance from the atomic column to this line is found. This is the (111) interplanar spacing at this point, as shown to the bottom right in Fig. 2a. Repeating this for every atomic column and its neighbor atom plane gives a 2-D map of the monolayer distances.

In the perovskites, a common structural change is unit cell doubling along a specific direction as a result of oxygen octahedral tilting [20]. Depending on the tilt pattern, this will result in the oxygen columns shifting. An example of this is shown in Fig. 2b, where there is an obvious oxygen superstructure along the black line signified by alternating distances between the oxygen columns in the [001]-direction. One useful way of quantifying this is finding how much the oxygen atom deviates from the centrosymmetric position in a cubic perovskite structure. This displacement, *D*, is found by determining the distance from an atom to the next (*N*) and the previous (*P*) atoms in the atom plane, and divide the difference (*N*−*P*) by 4. This gives the distance the oxygen atom deviates from a centrosymmetric position, and is shown in the inset in Fig. 2b. For a cubic perovskite structure *D* is 0, while for bulk LFO D is 0.39 Å.

### Atomic column shape

Having fitted every atomic column with an elliptical 2-D Gaussian (Eq. 1) where the rotation of the major axis from the vertical is one of the fit parameters (i.e., the direction of the $\sigma _{x,y}$ can change), one can extract information about the shape of the atomic column. This shape can reveal information about the structure parallel to the electron beam [19], and, in some materials, the position of light elements using the shape of the heavier atomic columns [13]. With the six parameters defined in Eq. 1, one can use the $\sigma _x$ and $\sigma _y$ to calculate the ellipticity for the atomic column which is a measure of how elongated the atomic column is. We define the ellipticity ($\epsilon$) as follows:

2 $$\begin{aligned} \epsilon = {\left\{ \begin{array}{ll} \frac{\sigma _x}{\sigma _y}, \quad \text {if } \sigma _x > \sigma _y\\ \frac{\sigma _y}{\sigma _x}, \quad \text {if } \sigma _y > \sigma _x\\ \end{array}\right. } \end{aligned}$$

giving an $\epsilon$ which is always greater or equal to 1. An $\epsilon$ of 1 would be a perfectly circular atomic column, while $\epsilon =2$ is an atomic column where one side is twice as long as the other side.

Similarly, one can use $\theta$ to find the direction of the ellipticity. We define this direction as $\rho,$

3 $$\begin{aligned} \rho = {\left\{ \begin{array}{ll} \theta , \quad &\text {if } \sigma _x > \sigma _y\\ \theta +\frac{\pi }{2}, \quad &\text {if } \sigma _y > \sigma _x\\ \end{array}\right. } \end{aligned}$$

which means that $\rho$ will be the angle between the positive x-axis and the longest $\sigma$. In addition, $\rho$ will always be between 0 and $\pi$ due to the symmetry of the 2-D Gaussian.

### The software implementation

This program is implemented in the Python (3.x) programming language, and both the source code and instructions on how to install it is available at http://atomap.org. It relies heavily on the fitting and modelling routines implemented in HyperSpy [39]. Currently, the program is optimized for analysing STEM-images of perovskite oxide materials projected along a $\langle110\rangle$ direction. However, it is trivial to adapt it for any structure as discussed below in "Adapting for other structures and projections". Extending the code should be easy, and requests for both new features and assistance in adapting for other structures are welcome on the issue tracker (linked from http://atomap.org/) or by e-mail to the corresponding author. The software and source code are distributed under the free and open source GNU General Public License v3.0.

The program is sorted into several classes: **atom_position**, **atom_plane**, **sublattice**, and **atom_lattice**. **atom_position** is the position of a single atomic column, and contains variables like position, $\sigma$, $\theta$, and other information about the shape of the atomic column. **atom_plane** contains all the **atom_positions** which belong to the same atomic plane. **sublattice** contains all the **atom_positions** and **atom_planes** belonging to the same sublattice, like the A-cations in Fig. 1. **atom_lattice** contains all the **sublattices**, so in Fig. 1, this would include the A-cations, B-cations, and oxygen sublattices. The **atom_lattice** class can be saved and loaded, saving all the **atom_position** parameters.

One current limitation is that the whole image given to the program must have a similar crystal structure. For example, a perovskite heterostructure shown in Figs. 4, 5, and 6 works fine, due to the structures being sufficiently similar. A perovskite oxide film grown on Si would, however, not work. Similarly, if there are any amorphous parts in the image, local bright features could be identified as atomic positions by the peak finding function. One simple solution is to crop the images, so only the same crystal structure is within the image given to the program. This could probably be automated, which would allow for automatic determination of regions with different structures. For example, in an aluminum alloy, one would be able to automatically figure out which regions are aluminum matrix and which are precipitates.

When fitting a single sublattice, the fitting is done on individual atomic column using a single 2D Gaussian. One obvious improvement would be to make a model containing all the atomic columns, and fitting them all simultaneously. While this is more computationally demanding, it could reduce the effects from neighboring atomic columns and, therefore, increase the accuracy of the fitting. The amount of improvement would be related to the degree of overlap between the atomic columns. With a very large separation between the atomic columns, this improvement would be zero or very small. With a very small separation, this improvement would be substantial. Quantifying the limits of fitting a single atomic column vs. including the neighboring atomic columns is interesting, but outside the scope of this work as the separation between the atomic columns was sufficiently large.

Atomap implements a version of this by removing the most intense sublattice before fitting the less intense sublattices. This is equivalent to having a model where the most intense atomic columns are fitted first, then locked, and afterwards fitting the second most intense atomic columns. Fitting 2D Gaussians to all atomic columns in a data set simultaneously will be experimented with in Atomap in the near future, using the aforementioned process to find reasonable initial values first.

### Adapting for other structures and projections

The first step in using the program is finding a value for the smallest atomic separation for the first sublattice. It is important to find a good value for this variable, as having either too many or too few atoms in the first sublattice will lead to the wrong repeating unit being identified. This then results in incorrect identification of the sublattice. In the example, discussed in "Initial positions and refinements", this value is half the separation between the A-cations in the [1$\overline{1}$0]-direction. The projected (110) spacing is 2.76 Å for STO. However, half the value (1.38 Å) did not work very well, which is caused by the scanning distortions and sample drift during image acquisition. By trial and error, a value slightly less than half the smallest separation was found to work reliably. For the STO in Fig. 1: approximately 1.3 Å. This value is used for the atom column separation in the peak finding function. For only fitting a single sublattice, this will be enough for the program to work.

Finding a second sublattice requires some *a priori* knowledge. For a perovskite oxide in the [1$\overline{1}$0]-projection (Fig. 1), one would specify that the atoms in the second sublattice are found between the atom columns in the (110)-atomic planes for the first sublattice.

These procedures are documented on the webpage (http://atomap.org/).

## Results and discussion

To test the processing method, data sets from epitaxial perovskite oxide heterostructures are used. First, the effects of imaging conditions and image registration on the results are tested. Then, a comparison is made between the method outlined in this article and GPA. Finally, the fitting of non-circular elliptical columns is tested.

All directions and atom planes are given in the pseudocubic coordinate system.

### Uncertainty and reducing scanning distortions

**Fig. 3:**
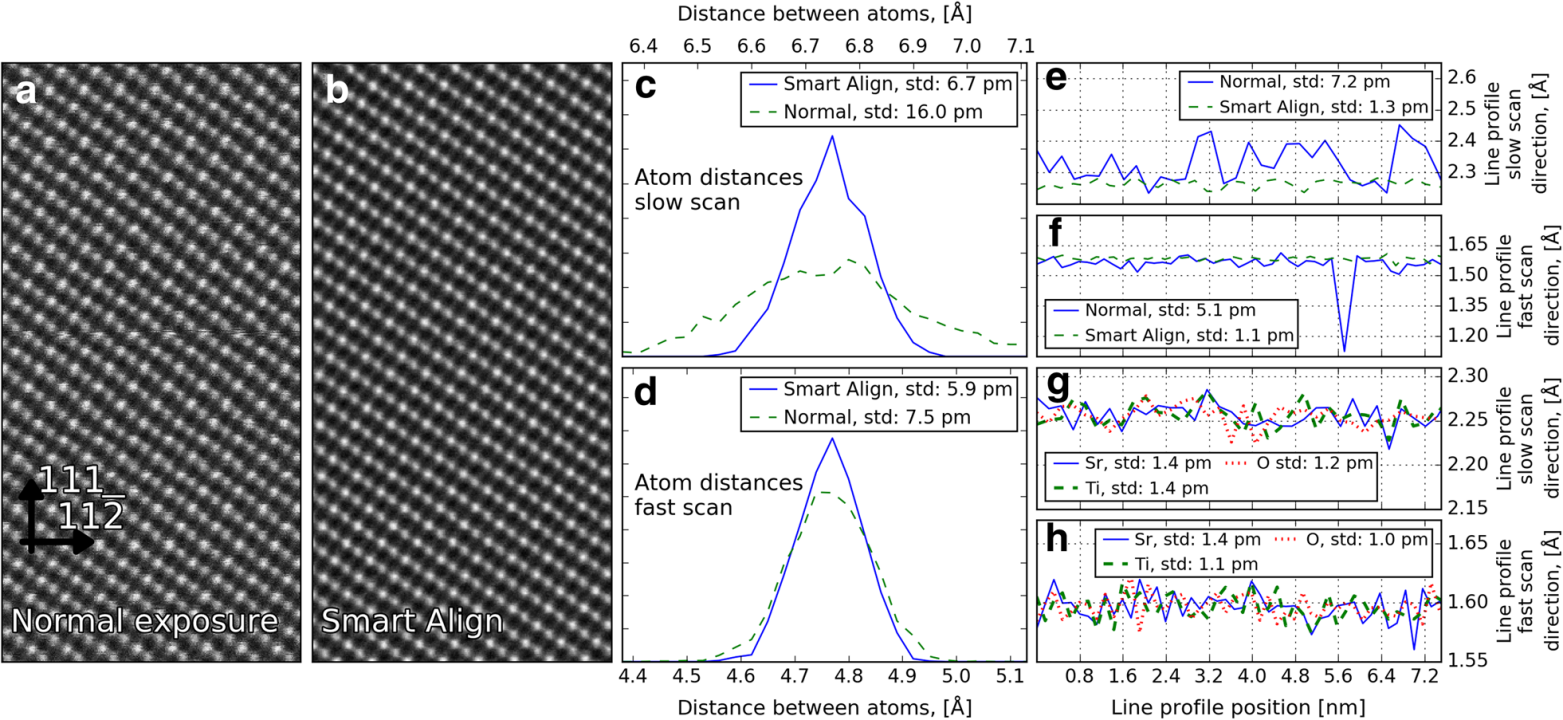
Quantifying the uncertainty in measurements of atomic spacings as a consequence of image acquisition methodology: **a** conventional HAADF-STEM image acquisition and **b** image constructed from a rapidly acquired image stack by rigid and non-rigid registration. Distribution of distances between Sr atoms along the **c** slow scan direction ([111]), and **d** fast scan direction ([$11\overline{2}$]), comparing the images from **a** and **b**. **e** Sr plane spacings along: **e** slow scan direction (111), and **f** fast scan direction ($11\overline{2}$). **g** and **h**: similar to **e** and **f**, but comparing uncertainties for the different sublattices from a different data set processed using Smart Align

It is clear that the reliability and the uncertainty of the peak fitting technique will be connected to how clearly the different atomic columns can be resolved. An important aspect of this is scanning distortions, as the electron probe is susceptible to both mechanical, electrical, and magnetic disturbances, both from noise within the microscope system, as well as extraneous disturbances from the surrounding environment, and these will have some influence, even in the best designed microscope rooms [40]. One way to reduce the effects of these distortions is to acquire several fast images of the same area, register them, and sum them afterwards [40]. In earlier studies, this was simply performed by rigid registration of the images, which takes out the effect of any drift, and simply averages out any local distortions (at the cost of a slight deterioration of the resolution) [3]. A better approach is to perform rigid registration to remove the effects of drift, followed by non-rigid registration to remove the effects of short period instabilities in the scanning system [41, 42]. To investigate the effect of short acquisition and alignment, as opposed to recording a single scan at a longer dwell time, we analysed two different STEM-ADF images from the same session taken along the [1$\overline{1}$0] cubic direction of a sample of STO: (i) acquired as a single image using a pixel dwell time of 38.5 ${\upmu }\mathrm{s}$ (line synced) with the image shown in Fig. 3a. (ii) Acquired as an image stack of 20 images with dwell time of 2 ${\upmu }\mathrm{s}$ per pixel (i.e., almost the same total acquisition time per pixel) which is then processed using Smart Align [42]. This aligned stack is shown in Fig. 3b. Clearly, the aligned stack appears a little sharper and less noisy than the single long acquisition image, but to quantify the effects of this difference on atom spacing measurements, the images were quantified as set out above in "Finding distances between atomic columns", and some comparative results are shown in Fig. 3c–h. A distribution of distances between the atoms parallel to the [111] (vertical in image) and [$11\overline{2}$] (horizontal in image) crystallographic directions of STO is shown in Fig. 3c, d: these directions are parallel to the slow and fast scan directions, respectively. As these data are from a region of a pure STO sample far away from any interfaces, there should be no variations in the distance between the planes. Therefore, these plots should be essentially a measure of the uncertainty in the measurements, which we define as one standard deviation in the spread of the distances. The difference between the single scanned image and the Smart Align image is very obvious, especially for the slow scan direction. In this case, the uncertainty is reduced from $\approx$16 p.m. to 7 p.m. For the fast scan direction, the reduction in uncertainty is much less: from 7.5 to 5.9 p.m.

Similar measurements for distances between the atomic planes in the slow and fast scan directions are shown in Fig. 3e and f, respectively. Compared to c and d, this is in effect averaging over many atomic columns. The uncertainty in the slow scan direction for the single scanned image is clearly much larger, and comparing the normal and Smart Align data sets, there is a massive reduction in the uncertainty from about 7 p.m. to 1 p.m. For the fast scan direction, the uncertainty in the single scan image is less, as might be expected, although there is a big negative spike at one point, which is typical for the kind of local disturbance that often arises in atomic resolution images. In this case, the uncertainty is reduced from about 5 p.m. to 1 p.m. Thus, it should be possible using non-rigid registration to make measurements of A-site (or other heavy, bright atom) plane spacings with about 1 p.m. precision.

One interesting effect is the decrease of uncertainty between the individual atomic columns and atomic planes for the single scanned image and the Smart Align image. For the fast scan atomic column uncertainty there is a small improvement for the aligned stack (7.5 to 5.9 p.m.), while for the atomic plane uncertainty there is a much larger improvement (5 to 1 p.m.). This is most likely due to the scanning distortions being correlated when acquiring a single scanned image using line synchronization. When acquiring an image stack with a short pixel dwell time of 2 ${\upmu }\mathrm{s},$ these scanning distortions are much less correlated. Since finding the distances between the atom planes consists of averaging the positions of many atomic columns, these uncorrelated local distortions in the image stack are averaged out.

Finally, the interplanar spacings for the different sublattices are shown in Fig. 3g, h. Here, a similar STO Smart Align ADF data set has been used to estimate the A- and B-cation uncertainty levels, and an ABF data set has been used to estimate the oxygen uncertainty levels. The average distances between the interplanar spacings are calculated for the A-cations, B-cations, and oxygen. For all atom types in both fast and slow scan directions, the uncertainty is about 1.0–1.4 p.m.

In view of the fact that the non-rigid registration method gives clearly superior results, all data sets in the following sections are acquired as image stacks and processed using Smart Align, and then processed using the method outlined in "Initial positions and refinements".

### Strain analysis: comparison to GPA

**Fig. 4:**
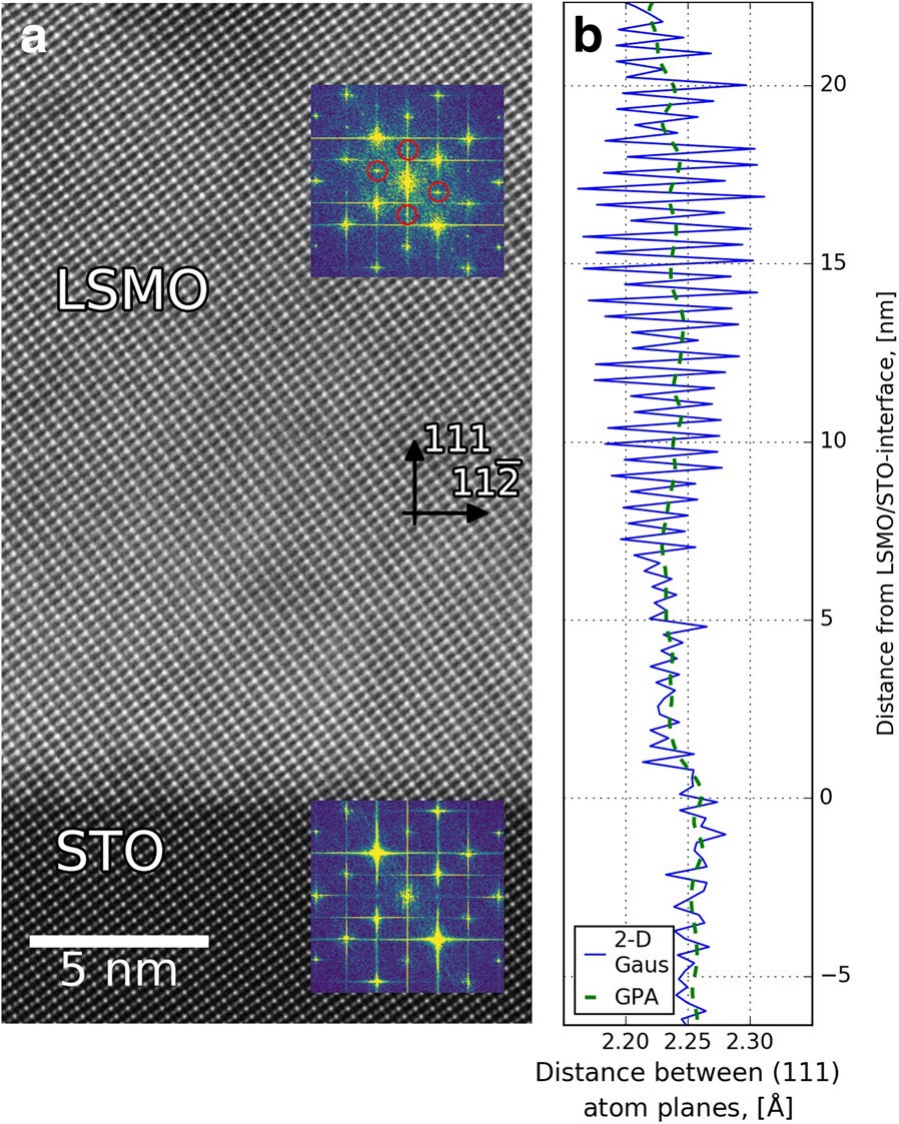
Comparison of geometrical phase analysis (GPA) and the method outlined in this work for determining the out-of-plane lattice parameter. **a** STEM-HAADF image of an LSMO film grown on STO-(111). *Top inset* is an FFT of the LSMO region, showing a clear superstructure compared to the STO substrate FFT in the *lower inset*. **b** Average interplanar distance for the A-cation (111) atom planes as a function of distance from the LSMO/STO interface. The *blue solid line* shows the result from the method outlined in this paper, and the *dashed green line* from GPA

As stated in the introduction, GPA is a commonly used technique for characterizing distances between atomic columns in atomic resolution (S)TEM images [21]. Figure 4 shows a comparison between GPA and the method explained in this work, for an epitaxial LSMO/STO-(111) heterostructure. Figure 4a shows a STEM-ADF image and FFTs from the LSMO (top inset) and STO (bottom inset). The fast Fourier transform (FFT) of the LSMO region has extra $\left\{ \frac{1}{2}\frac{1}{2}\frac{1}{2}\right\}$ spots (circled in red) which are not present in the STO region. These superstructure spots indicate doubling of the unit cell along the [111]- and [11$\overline{1}$]-directions in the LSMO. GPA is performed using the {111} and {11$\overline{2}$} FFT spots utilizing the STO substrate far away from the interface as a reference. These data are then summed in the [111]-direction, giving the average out-of-plane distance as a function of distance from the LSMO/STO interface (Fig. 4b, green dashed line). Using the method explained in "Finding distances between atomic columns", the average distance between the A-cation monolayers in the [111]-direction is calculated as a function of the distance from the interface (Fig. 4b, blue line). Comparing the two line profiles, they both show the same general trends: larger lattice size in the STO, and smaller in the LSMO, with a slight lattice size increase at about 8 nm into the film. However, the unit cell doubling observed in the FFT is only visible in the real space method. This is due to the choice of the mask in the GPA, since the one used to get the data in Fig. 4 did not include the superstructure spots. It could be possible to use the $\left\{ \frac{1}{2}\frac{1}{2}\frac{1}{2}\right\}$ spots to do the GPA, instead of the {111} and {11$\overline{2}$}. This could show the presence of the unit cell doubling, but the regions where these peaks are not present would not yield any information, for example, the STO substrate and the regions in the LSMO film without the unit cell doubling.

### Mapping oxygen octahedral tilting

**Fig. 5:**
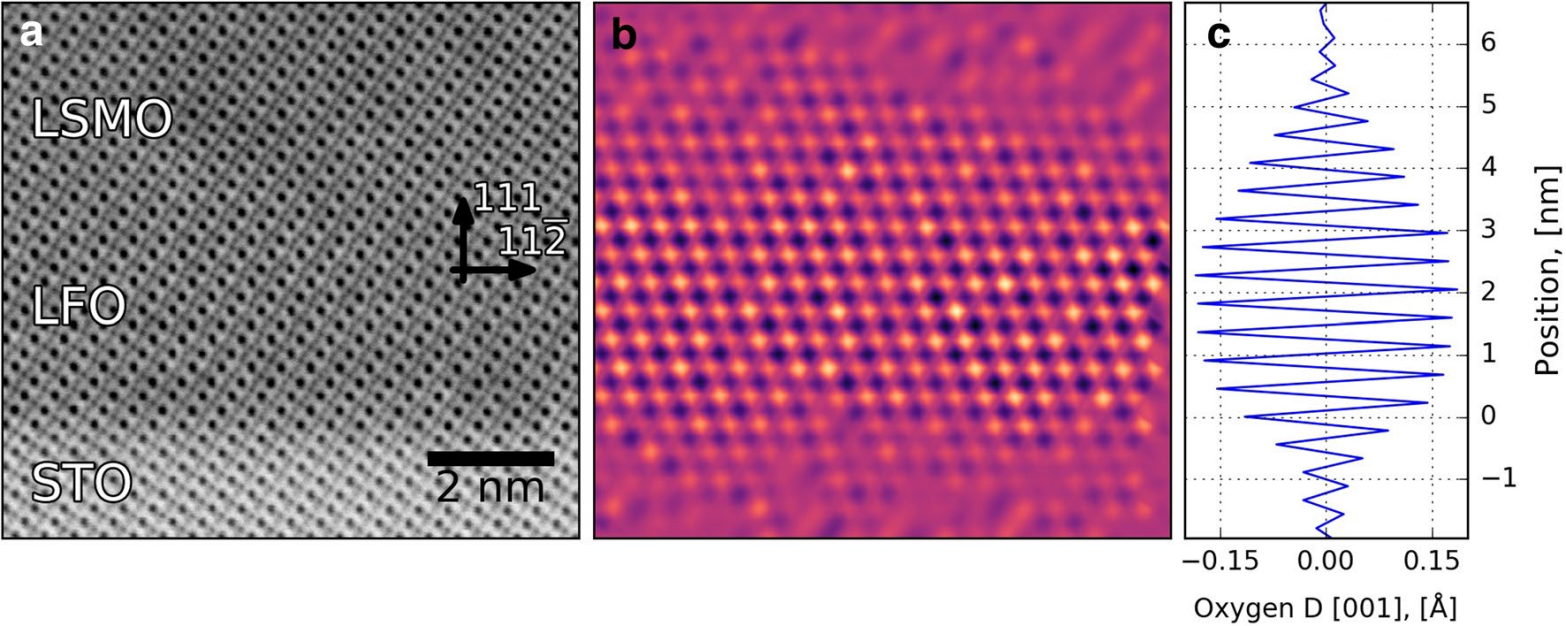
Finding the displacement, D, of oxygen atomic columns in the [001]-direction, compared to centrosymmetric positions in a cubic structure. **a** STEM-ABF image of LSMO/LFO/STO-(111) heterostructure. **b** Map of the deviation of oxygen positions from those for a primitive cubic perovskite calculated using the method outlined in "Finding distances between atomic columns". **c** Average value of the displacement for each plane as a function of distance from the LFO/STO-interface

Figure 5a shows an ABF image of the LSMO/LFO/STO-(111) heterostructure, where there is a “zig-zag” pattern of the oxygen columns along the [110]-direction in the LFO layer. This displacement from the centrosymmetric position can be quantified using the method discussed in "Finding distances between atomic columns". An example of this is shown in Fig. 5b, where the [001]-direction displacement is calculated for all the oxygen columns. The displacement in the LFO layer takes the form of an alternating long and short displacement in the [001]-direction. To give a better visualization, the displacement is set to zero at the A-cation positions, leading to the checkerboard pattern shown in Fig. 5b. An average of the displacements as a function of distance from the LFO/STO interface is shown in Fig. 5c. This shows both a clear superstructure and the variation in the displacement across the film. This can then be used to infer parameters, such as the octahedral tilting pattern and bond angles.

One important caveat with using ABF imaging is the relationship between real and measured atom column positions is not always intuitive [43]. For example, if the sample is slightly tilted off the zone axis, the atomic positions can shift. This effect varies with respect to thickness, atomic number, collection angle, and convergence angle [43]. Thus, to properly analyse the atomic positions, image simulations coupled with careful considerations of the experimental parameters are required. However, the measured atomic positions are still useful as the first approximations, even if further work with image simulations is required to ensure that the conclusions are robust.

### Ellipticity

**Fig. 6:**
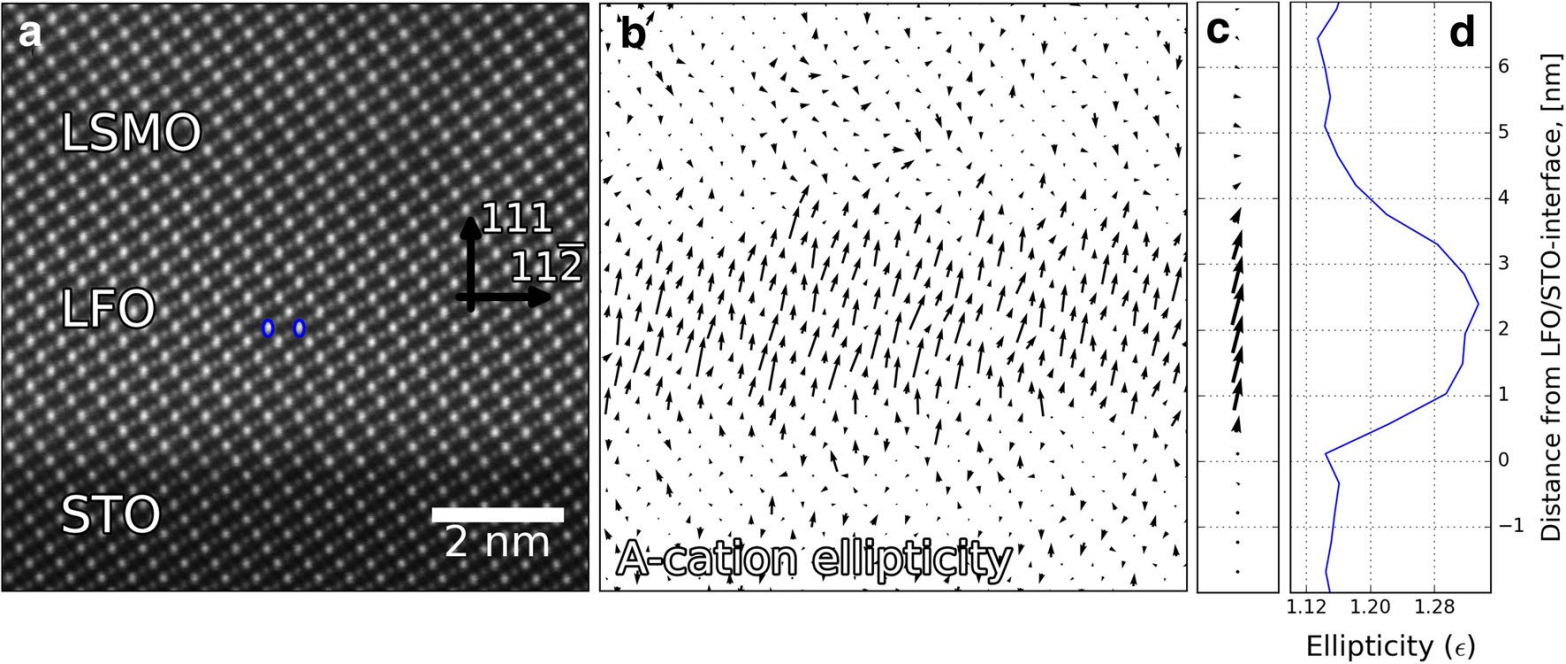
Mapping ellipticity ($\epsilon$) and rotation ($\rho$) of A-cation atomic columns in the LSMO/LFO/STO-(111) heterostructure. **a** STEM-ADF image of the heterostructure. There is a clear elongation of the A-cation atomic columns in the LFO layer (*blue ellipses*), compared to the STO and LSMO. **b** $\epsilon$ and $\rho$ of the individual A-cations plotted as *arrows*. **c** Same as in **b**, but summed as a function of distance from the LFO/STO interface. **d** $\epsilon$ as a function of distance from the LFO/STO interface.

As discussed in "Atomic column shape", one can also use the shape of the atomic columns to extract structural information [12]. An example of this is seen in Fig. 6a, which shows A-cation ellipticity in the LSMO/LFO/STO-(111) heterostructure imaged using STEM-ADF. In the LFO layer, there is a clear elongation of the A-cation sites, annotated with the blue ellipses. By fitting elliptical 2-D Gaussians (Eq. 1) to every A-cation, this elongation can be quantified with $\epsilon$ (Eq. 2) and $\rho$ (Eq. 3). Where $\epsilon$ is $\frac{\sigma _{\rm Longest}}{\sigma _{\rm Shortest}}$, and $\rho$ is the angle between the positive* x*-axis and $\sigma _{\rm Longest}$. Having these values for every A-cation, one can visualize these in a vector plot (Fig. 6b). Here, the length of the arrows is given by $\epsilon$, and the direction by $\rho$. There is a clear difference in the LFO layer, which has an elongation close to the [111]-direction. This is even more apparent with the vector plot in Fig. 6c and the $\epsilon$ plot in Fig. 6d, which takes the average of the data as a function of distance from the interface. The ellipticity is constant in the STO substrate until the LSMO/STO interface, where it increases to its maximum about 2 nm into the film, and decays gradually in the LSMO film to the same level as in the STO substrate.

As discussed in "Atomic column shape", there are many variables which can affect ellipticity of the atomic columns. Therefore, for the vector plots in Fig. 6b, c, a systematic average $\epsilon$ and $\rho$ “background” have been subtracted. This noise level was determined from the STO substrate ($\approx$0.12 $\epsilon$ in the [111]-direction), where the ellipticity should be 1. This systematic background is most likely due to a variety of factors: sample drift, astigmatism, misalignment, having the sample slight off-axis, and residual scanning distortions. Especially, the latter is present in this data set, as horizontal “streaks” visible in some of the atomic columns (Fig. 6a). These are still present, even though the image was made by averaging an image stack. This is due to the fast scan being in the same direction for all the images in the image stack. One solution is rotating the fast scan direction by 90° in every second image in the image stack, which will average out this scanning distortion. Another effect can be the 2-D Gaussian fitting itself, where overlap from the neighboring atomic columns can influence the fitting. For example, in Fig. 6, the B-cations might add a slight component towards the [001]-direction.

## Conclusion

In summary, we have developed a free and open source software package for automatically quantifying the positions and shapes of atomic columns in atomic resolution STEM-images. By utilizing a model-based approach with 2-D Gaussians, the most intense atomic columns are subtracted, and all the sublattices in a STEM image can be analysed separately. Furthermore, the projected average 2-D atomic arrangement is automatically extracted by finding the atomic planes with the largest spacings. This is used to organize the atomic columns in atomic planes, which facilitates the analysis of interplanar distances. Using images where the scanning distortions had been reduced using non-rigid registration, a precision of 6p.m. could be obtained for distances between single atomic columns, and 1.2 p.m. for distances between atomic planes.

The software has been tested on HAADF and ABF STEM images of perovskite oxide heterostructures. The displacement of oxygen columns in ABF images was quantified. Using elliptical 2-D Gaussians, the projected shape of the A-cation atomic columns was extracted. The software should be easily adaptable for any atomic resolution STEM image, as long as the atoms are clearly resolved.

## Abbreviations

STEM: scanning transmission electron microscopy
ADF: annular dark field
HAADF: high angle annular dark field
ABF: annular bright field
GPA: geometrical phase analysis
FFT: fast Fourier transform
PCA: principal component analysis
STO: SrTiO$$_3$$
LSMO: La$_{0.7}$Sr$_{0.3}$MnO$_3$
LFO: LaFeO$$_3$$
